# A Smartphone-Based Platform Assisted by Artificial Intelligence for Reading and Reporting Rapid Diagnostic Tests: Evaluation Study in SARS-CoV-2 Lateral Flow Immunoassays

**DOI:** 10.2196/38533

**Published:** 2022-12-30

**Authors:** David Bermejo-Peláez, Daniel Marcos-Mencía, Elisa Álamo, Nuria Pérez-Panizo, Adriana Mousa, Elena Dacal, Lin Lin, Alexander Vladimirov, Daniel Cuadrado, Jesús Mateos-Nozal, Juan Carlos Galán, Beatriz Romero-Hernandez, Rafael Cantón, Miguel Luengo-Oroz, Mario Rodriguez-Dominguez

**Affiliations:** 1 Spotlab Madrid Spain; 2 Servicio de Microbiología Hospital Universitario Ramon y Cajal Madrid Spain; 3 Servicio de Geriatría Hospital Universitario Ramon y Cajal Madrid Spain; 4 Instituto Ramón y Cajal de Investigación Sanitaria (IRYCIS) Madrid Spain; 5 Biomedical Image Technologies ETSI Telecomunicación Universidad Politécnica de Madrid Madrid Spain; 6 CIBER en Epidemiología y Salud Pública (CIBERESP) Instituto de Salud Carlos III Madrid Spain; 7 CIBER en Enfermedades Infecciosas (CIBERINFEC) Instituto de Salud Carlos III Madrid Spain

**Keywords:** rapid diagnostic test, artificial intelligence, AI, telemedicine platform, COVID-19, rapid test, diagnostics, epidemiology, surveillance, automatic, automated, tracking

## Abstract

**Background:**

Rapid diagnostic tests (RDTs) are being widely used to manage COVID-19 pandemic. However, many results remain unreported or unconfirmed, altering a correct epidemiological surveillance.

**Objective:**

Our aim was to evaluate an artificial intelligence–based smartphone app, connected to a cloud web platform, to automatically and objectively read RDT results and assess its impact on COVID-19 pandemic management.

**Methods:**

Overall, 252 human sera were used to inoculate a total of 1165 RDTs for training and validation purposes. We then conducted two field studies to assess the performance on real-world scenarios by testing 172 antibody RDTs at two nursing homes and 96 antigen RDTs at one hospital emergency department.

**Results:**

Field studies demonstrated high levels of sensitivity (100%) and specificity (94.4%, CI 92.8%-96.1%) for reading IgG band of COVID-19 antibody RDTs compared to visual readings from health workers. Sensitivity of detecting IgM test bands was 100%, and specificity was 95.8% (CI 94.3%-97.3%). All COVID-19 antigen RDTs were correctly read by the app.

**Conclusions:**

The proposed reading system is automatic, reducing variability and uncertainty associated with RDTs interpretation and can be used to read different RDT brands. The web platform serves as a real-time epidemiological tracking tool and facilitates reporting of positive RDTs to relevant health authorities.

## Introduction

To control COVID-19 pandemic, timely and accurate early-detection strategies of SARS-CoV-2 infections have been critical to slow down the spread of the virus. The use of rapid diagnostic tests (RDTs), both for detection of antibodies and antigens, has contributed to improve COVID-19 testing capacity, reducing costs of diagnosis, and allowing for fastest results [1]. First, COVID-19 RDTs were intended to be used just by professional health workers who have extensive experience in the use of this tool for different infectious diseases [2,3]. Later, multiple health ministries approved home testing kits, improving the accessibility to testing and taking pressure off health institutions. Nevertheless, self-testing strategies have some limitations; the general population is not familiar with the use of RDTs; and a minimum training is needed for sampling, testing, and result interpretation. Furthermore, as it has been seen during the latest waves [4], many results go unreported, impairing posttesting counseling and epidemiological surveillance.

**Figure 1:**
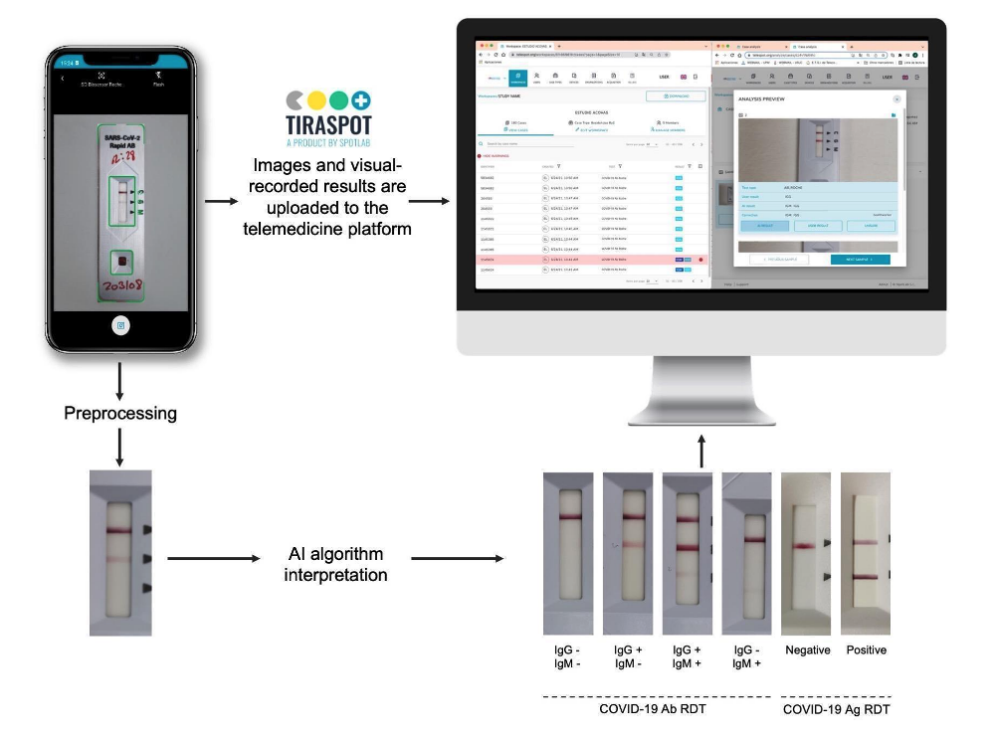
TiraSpot system is composed of (1) a mobile app for test digitization and result recording, (2) an artificial intelligence (AI) model for rapid diagnostic test (RDT) result interpretation, and (3) a web platform where all collected data can be visualized, allowing for result corrections in the cases in which a discrepancy exists between AI and user interpretation. Ab: antibody; Ag: antigen.

Combining RDTs with digital tools, artificial intelligence (AI) and mobile health approaches can help standardize result interpretation and facilitate immediate reporting and monitoring of results [5]. Several works have been proposed to automatically interpret photographs of RDTs using different image processing approaches, from classical methods, such as morphology-based methods, to more sophisticated machine learning or deep learning methods [6-21]. Nevertheless, these approaches are not capable of handling 2-band and 3-band RDTs indistinctly, are not connected to a cloud platform that enables the collection of mass screening results, and many require additional hardware. In this paper, we describe the development and field validation of a mobile-based tool (exhaustively tested with a variety of phone models and different lighting conditions) that could be used with any smartphone for reading and reporting multiple types of SARS-CoV-2 RDTs and is connected to a real-time epidemiological monitoring web platform (Figure 1).

## Methods

### Procedure

This study was divided into 2 phases: first, the training and validation of an AI algorithm for the automatic interpretation of RDTs; second, 2 field studies to assess the performance of the AI-based system for reading both COVID-19 antibody and antigen RDTs in real-world scenarios.

### Ethics Approval

Ethics approval for the study was obtained from the Clinical Research Ethics Committee of the Ramón y Cajal University Hospital (127/21).

### Algorithm Training and Validation Data Set

Ensuring standardized image acquisition is a key step in developing robust AI algorithms. With this purpose, all inoculated RDTs were digitized using the TiraSpot mobile app (Spotlab), which guarantees image quality and correct positioning of RDTs in the image by using a simple augmented reality system that displays a mask with the exact geometry of a given RDT in the screen of the smartphone, helping users to correctly align the RDT before making the picture (screenshot of the mobile app is presented in Figure 1). Each RDT brand has its own mask that guides the user to take a standardized picture. In addition, after the picture is taken, the user is presented with the picture and asked to confirm that it is aligned and on focus. If the user rejects the picture, the user is allowed to take another one. The mobile app also allows users to record sample metadata, which together with the images and their initial visual interpretation are uploaded to the cloud platform. To gain robustness and generalizability, a total of 11 different smartphone models, ranging from low- to high-range devices, were used in this study.

**Figure 2:**
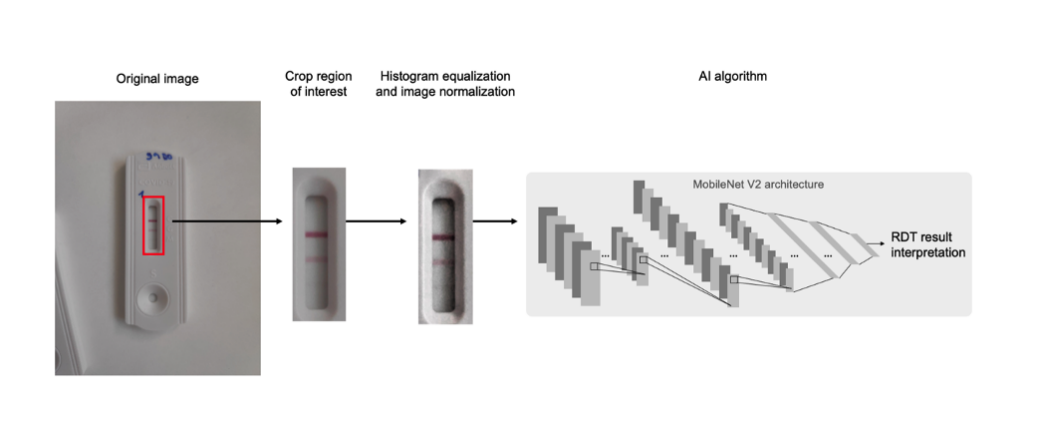
Image processing pipeline. Original image acquired with the TiraSpot app is first cropped to extract region of interest. The cropped image is then preprocessed and introduced to a convolutional neural network, which predicts rapid diagnostic test (RDT) result. AI: artificial intelligence.

An AI algorithm was developed to predict test results based on a picture of the RDT. With this purpose, the image is first preprocessed by cropping the original image to extract a region of interest that contains the relevant part of the picture (strip of the RDT). Then, image normalization and contrast enhancement (Contrast Limited Adaptive Histogram Equalization method) were applied to highlight faint bands. Finally, the processed region of interest is introduced into a convolutional neural network (MobileNet V2 [22]), which then predicts the test result (Figure 2).

For generating the training image data set, 12 human sera from patients with positive SARS-CoV-2 polymerase chain reaction tests (infected between March and May 2020) and with a positive enzyme-linked immunosorbent assay (ELISA) test were used. Each serum sample was serially diluted with reference human sera (H5667; Sigma-Aldrich) until it reached a negative result when inoculated in a COVID-19 antibody test. Each dilution was tested in 3 replicates for each of the 3 brands tested (ie, 2019-nCoV IgG/IgM Rapid Test Cassette, Hangzhou AllTest Biotech Co., Ltd.; Panbio COVID-19 IgG/IgM Rapid Test Device, Abbott; and UNscience COVID-19 IgG/IgM Rapid Test, Wuhan UNscience Biotechnology Co., Ltd.), resulting in 433 RDTs inoculated (61 positive for both IgG and IgM; 166 positive for IgG and negative for IgM; 43 negative for IgG and positive for IgM; and 164 negative for both IgG and IgM). Additionally, 12 COVID-19 antigen RDTs (Panbio COVID-19 Ag Rapid Test Device, Abbott; 6 positive and 6 negative) were also included to train the algorithm to read not only 3-band tests (such as the COVID-19 antibody tests used in this study) but also 2-band RDTs, such as COVID-19 antigen tests. The entire training data set consisted of 3614 images.

For collecting the independent validation data set, 240 human sera samples independent from the ones used for training were used to inoculate 720 COVID-19 antibody RDTs (each serum was tested in triplicate using the aforementioned brands). The samples were selected ensuring all possible results are well represented along the data set (108 positive for both IgG and IgM; 321 positive for IgG and negative for IgM; 27 negative for IgG and positive for IgM; and 264 negative for both IgG and IgM).

Each RDT was visually read by multiple observers (3 to 5), and the ground truth was established as the majority result from total analyzers. All sera samples were collected between May and June 2020.

### Field Validation Studies

The workflow for the field studies was as follows: a health professional digitized the RDTs by using the app and was asked for recording the visual interpretation of the test result; images were uploaded to the cloud platform and processed by the AI algorithm; and discrepancies between the interpretation made by the health professional and that obtained by the algorithm were subsequently reviewed by an external health professional through the platform.

The first field study used the system as part of a seroprevalence study conducted in two nursing homes in Madrid, Spain. A total of 172 vaccinated health care personnel were included in this study; a finger-prick blood sample was taken from them and inoculated into SARS-CoV-2 Rapid Antibody Test (Roche). A trained nurse digitized the RDTs and recorded their results using the app.

The second field validation study tested the system to read also COVID-19 antigen tests (ie, Panbio COVID-19 Ag Rapid Test Device, Abbot) composed of 2 bands (ie, control and test). This study was carried out at the emergency department of the Ramón y Cajal Hospital in Madrid, Spain, where 96 individuals’ nasal swabs were inoculated in antigen tests and digitized by experienced health professionals using the app.

All images were acquired in very diverse real-world conditions involving different users, including different environmental illuminations (eg, different lighting color temperatures and a wide range of lighting), and using different smartphone models that ranged from low- to high-range devices. This was done with the purpose of developing and validating the robustness of the algorithm that may change in real life.

## Results

### AI Algorithm Training and App Validation

All images acquired with the app were uploaded to a cloud platform, where the AI algorithm processed the photographs to predict the result interpretations. As shown in Table 1 (part 1), when comparing the visual interpretations (used as ground truth) against the AI algorithm, the performance was high for all brands of RDT tested, obtaining a mean sensitivity and specificity of 98% and 100%, respectively, for the detection of the IgG band; and a mean sensitivity and specificity of 80% and 89%, respectively, for the detection of the IgM band. No significant differences were found in algorithm performance between different smartphone models or across different lighting conditions, pointing out the robustness of the readout algorithm.

**Table 1:** Performance of the artificial intelligence algorithm for predicting rapid diagnostic test (RDT) results with respect to human visual reading in (1) the validation set, (2) the field study for reading antibody (Ab) RDTs, and (3) in the field study when reading antigen (Ag) RDTs.

Evaluation data				AUC^a^ (95% CI)		Sensitivity (95% CI)		Specificity (95% CI)		Tests, n		
										Negative		Positive
**1: RDT manufacturer (Ab) and band**												
	**Abbott**											
		IgG	99.5 (98.7-100)		96.4 (94.1-98.8)		100 (100-100)		94		145	
		IgM	92.5 (85.4-99.6)		80.8 (75.8-85.8)		90.7 (87.0-94.3)		184		55	
	**UNScience**											
		IgG	100 (100-100)		100 (100-100)		100 (100-100)		100		140	
		IgM	89.5 (83.7-95.2)		80.0 (74.9-85.1)		88.6 (84.6-92.6)		214		26	
	**AllTest**											
		IgG	99.8 (99.4-100)		97.9 (96.1-99.7)		100 (100-100)		96		144	
		IgM	90.6 (85.0-96.1)		79.6 (74.5-84.7)		86.0 (81.6-90.4)		186		54	
	**Global**											
		IgG	99.8 (99.5-100)		98.1 (97.1-99.1)		100 (100-100)		290		429	
		IgM	90.8 (87.4-94.3)		80.0 (77.1-82.9)		89.0 (86.2-90.9)		584		135	
**2: RDT manufacturer (Ab) and band**												
	**Roche**											
		IgG	100 (100-100)		100 (100-100)		94.4 (92.8-96.1)		18		154	
		IgM	99.6 (96.0-100)		100 (100-100)		95.8 (94.3-97.3)		166		6	
**3: RDT manufacturer (Ag) and band**												
	**Abbott**											
		Test	100 (100,100)		100 (100,100)		100 (100,100)		68		28	

^a^AUC: area under the curve.

### Validation in Real-world Scenarios

From the 172 RDTs used in this study (5 positive for both IgG and IgM; 149 positive for IgG and negative for IgM; 1 negative for IgG and positive for IgM; and 17 negative for both IgG and IgM), we only found 9 discrepancies between test result interpretations made by health professionals and those made by the AI algorithm. From these 9 cases, 2 were incorrectly classified by the algorithm due to an incorrect image acquisition with the app. The remaining discrepant cases were further reviewed by a second professional, and the AI-based system allowed for the detection and modification of the result with respect to the initial health professional interpretation in 4 cases by confirming the result predicted by the algorithm.

The overall performance of the algorithm with respect to the ground truth is shown in Table 1 (part 2). It should be noted that the performance of the system is high even when used with an RDT different from those used for training the algorithm, suggesting its potential use with any RDT on the market. The slight disparity in the performance of IgM band identification in antibody RDTs between the validation set and this field study may be explained by the presence of very faint signals that were almost invisible in the photographs.

Regarding the second field study for reading COVID-19 antigen RDTs, we found that all tests used and digitized using the TiraSpot app (ie, 58 negative and 30 positive) were correctly interpreted by the proposed system (Table 1, part 3), demonstrating that the system can also be applied for reading 2-band (ie, control and test) and 3-band (ie, IgG, IgM, and control) tests.

## Discussion

We described the usefulness of an app for reading and result interpretation of lateral flow RDTs for SARS-CoV-2 testing. The results are sent to a cloud platform that allows for case identification and confirmation, quality control, and real-time monitoring.

Our AI algorithm demonstrates excellent performance, especially in prospective validation of real-life scenarios and for both antibody and antigen detection tests. The algorithm performed as well in RDT brands that were not used at all for training purposes, making the solution suitable for other RDTs, including other diseases. Compared with previous studies [6-21], our system is able to identify individual bands of the RDTs, allowing for complex result reading and sending them in real-time to a cloud platform. A requirement and limitation of the proposed system is the correct acquisition of the image (acquisition error in the field studies was <0.8%).

In conclusion, the use of TiraSpot (Figure 1) is a useful tool for reporting, real-time monitoring, and quality control, as the results can be reviewed by specialists when needed. This is especially important in contexts where massive testing is to be done and the likelihood of subjectivity and errors in the interpretation of the result is higher. It is also important in the validation of self-diagnostic tests performed by untrained users, as it avoids the loss of information in case the user does not report it, and it provides an efficient system to confirm and report data, which has been a key challenge during the latest pandemic waves.

## Abbreviations

AI: artificial intelligence
RDT: rapid diagnostic test

## Data Availability

The raw data supporting the results of this study will be made available upon reasonable request from the corresponding author.
